# Supplementary material for: The unstructured linker arms of MutL enable GATC site incision beyond roadblocks during initiation of DNA mismatch repair
Source: Nucleic Acids Res. 2019 Oct 10;47(22):11667–80. doi: 10.1093/nar/gkz834 (PMC6902014; doi:10.1093/nar/gkz834)
Supplement: gkz834_Supplemental_File [file gkz834_supplemental_file.pdf]

Supplemental Figures Mardenborough et al.

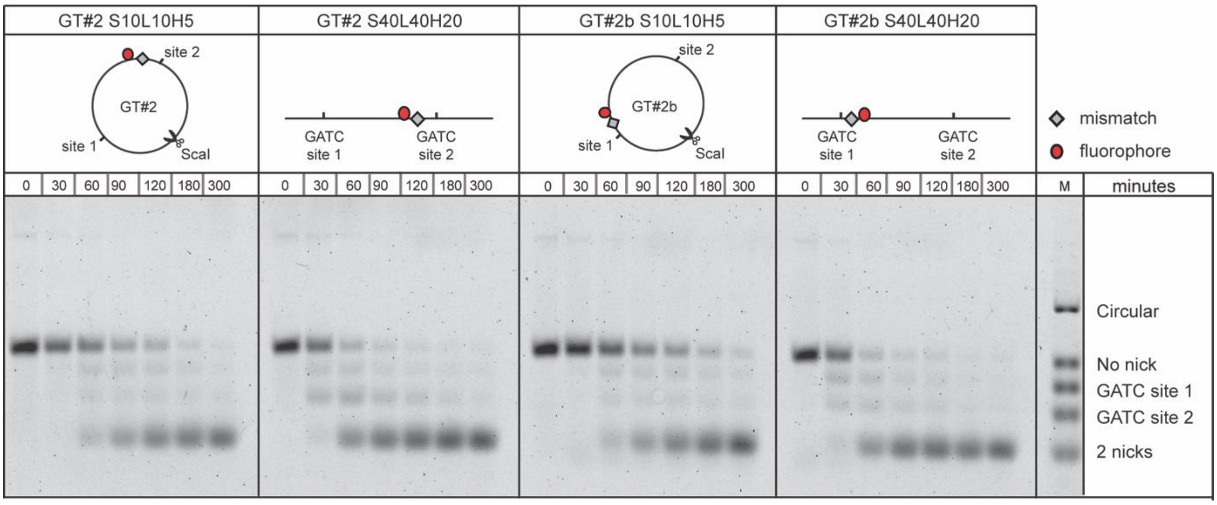

Supplemental Figure S1: GATC site preference on linear but not on circular DNA.

Time courses of GATC site incision by MutS, MutL and MutH (protein concentrations as indicated at the top) on 0.5 nM GT#2 (2 panels on the left) and GT#2b (2 panels on the right). Reactions on circular DNA were stopped by heat inactivation followed by linearization with ScaI. Reaction products were separated using gel electrophoresis under denaturing conditions and visualized using the Alexa^{647} fluorophore.

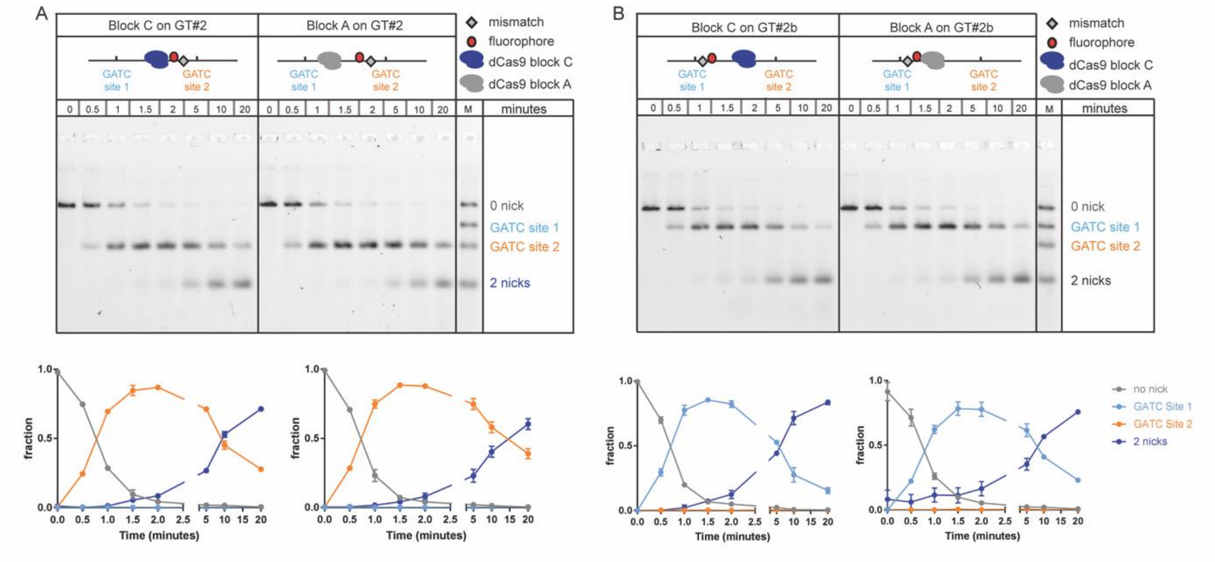

Supplemental Figure S2: dCas9 roadblock location does not influence bypass efficiency.

Time courses of GATC site incision by 50 nM MutS, 50 nM MutL and 25 nM MutH on 0.5 nM GT#2 (panel A) and GT#2b (panel B) with roadblocks at position C (blue) in the center of the substrate or position A (orange) close to GATC site 1. Reaction products were separated using gel electrophoresis under denaturing conditions and visualized using the Alexa^{647} fluorophore. Graphs show quantification of product fractions containing no nick (gray), a nick at GATC site 1 (light blue), a nick at GATC site 2 (orange) and 2 nicks (dark blue). Data points with error bars represent the mean values and range of three independent experiments.

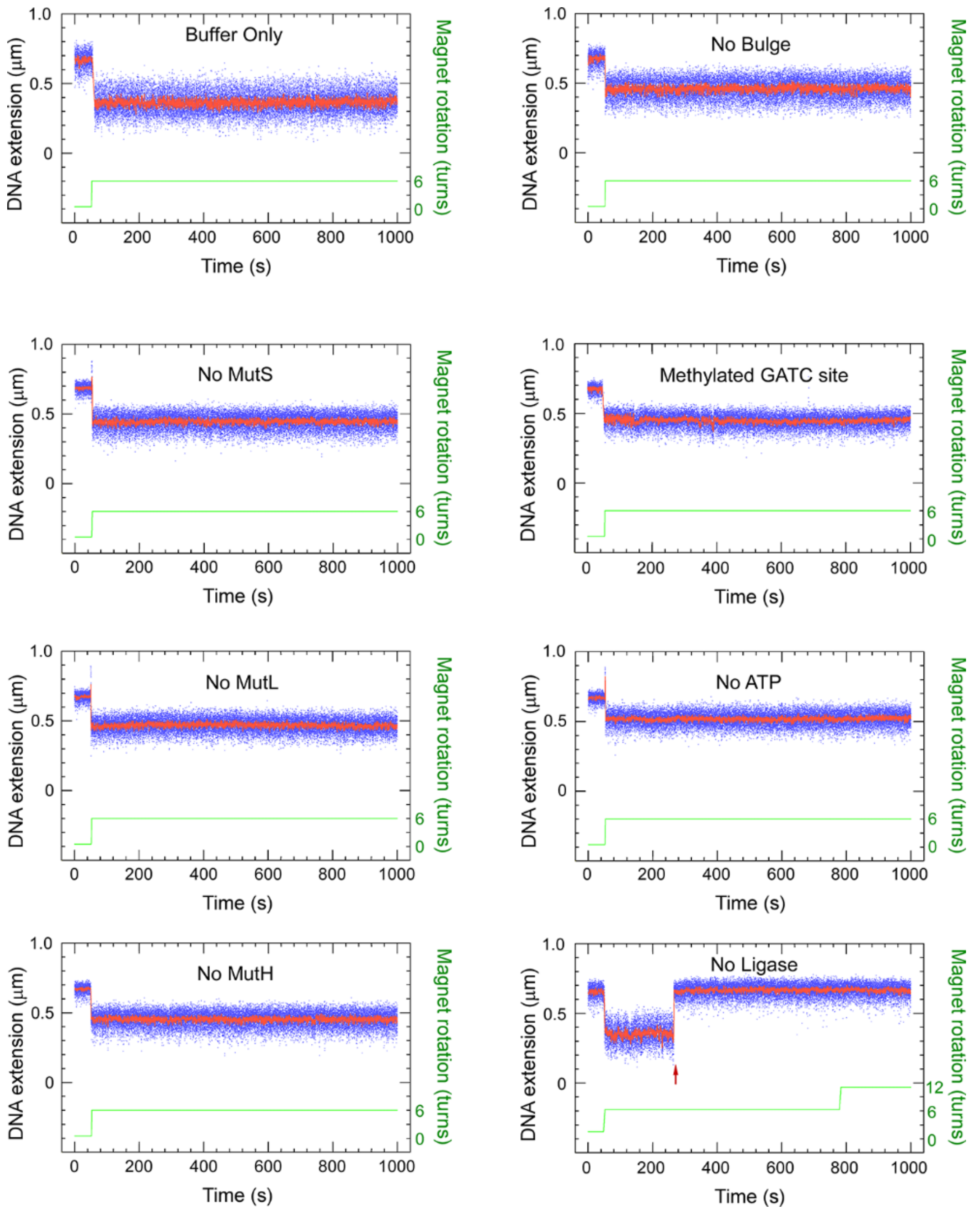

Supplemental Figure S3: control experiments for single-molecule MMR incision assay.

Time-traces obtained using supercoiled Rb-pREP4 construct bearing a bulge show that reaction buffer alone does not cause DNA incision, and that removing any one of MutS, MutL or MutH (as indicated) from the complete set of reaction components also abolished incision. Furthermore, DNA lacking a bulge is not a substrate for MMR (No bulge), nor are DNA molecules with a methylated GATC site (Methylated GATC site). Withholding ATP also abolished incision (no ATP). Finally, in the

absence of T4 DNA ligase the DNA could be incised but not supercoiled again (No Ligase). When present, reaction component concentrations were 2.5 nM MutS, 10 nM MutL, 10 nM MutH, and 0.1 mM ATP. Blue points show bead position sampled at 31 Hz, red points show raw data with ~1 s averaging. Green line represents the stepwise increase in supercoiling imposed on the DNA via the magnetic trap. Red arrow: incision event.

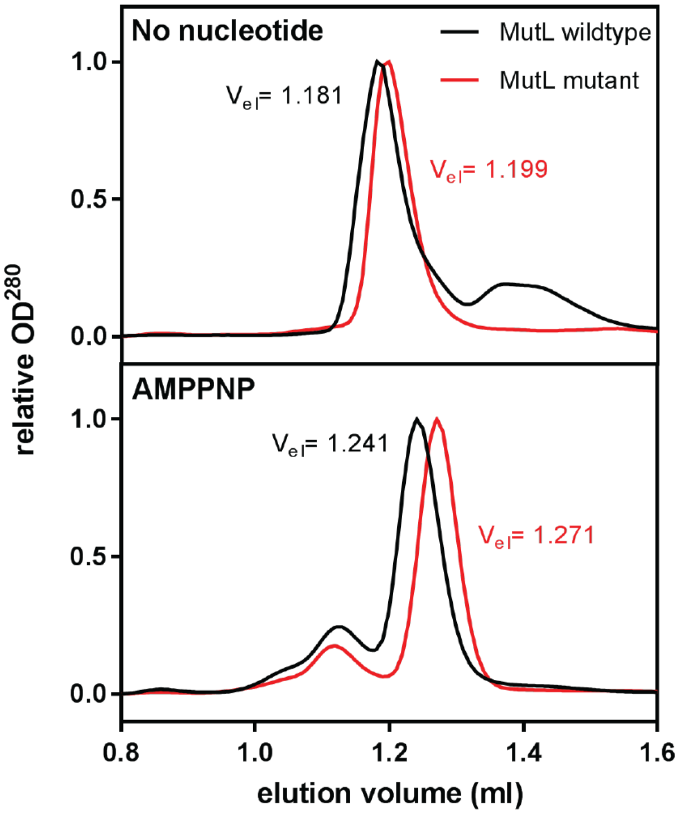

Supplemental Figure S4: The MutL linker mutant has a smaller hydrodynamic volume than wild type MutL in the nucleotide-free and -bound states.

Upper panel: Normalized size exclusion chromatography data of wild-type MutL (black) and MutLΔ4 (red) in open form. The increase in elution volume (V_{el}) indicates a smaller hydrodynamic volume for MutLΔ4 compared to wild type MutL. Lower panel: Wild-type and MutLΔ4 MutL in the closed state, induced by the presence of nucleotide AMPPNP show a similar difference in hydrodynamic volume.

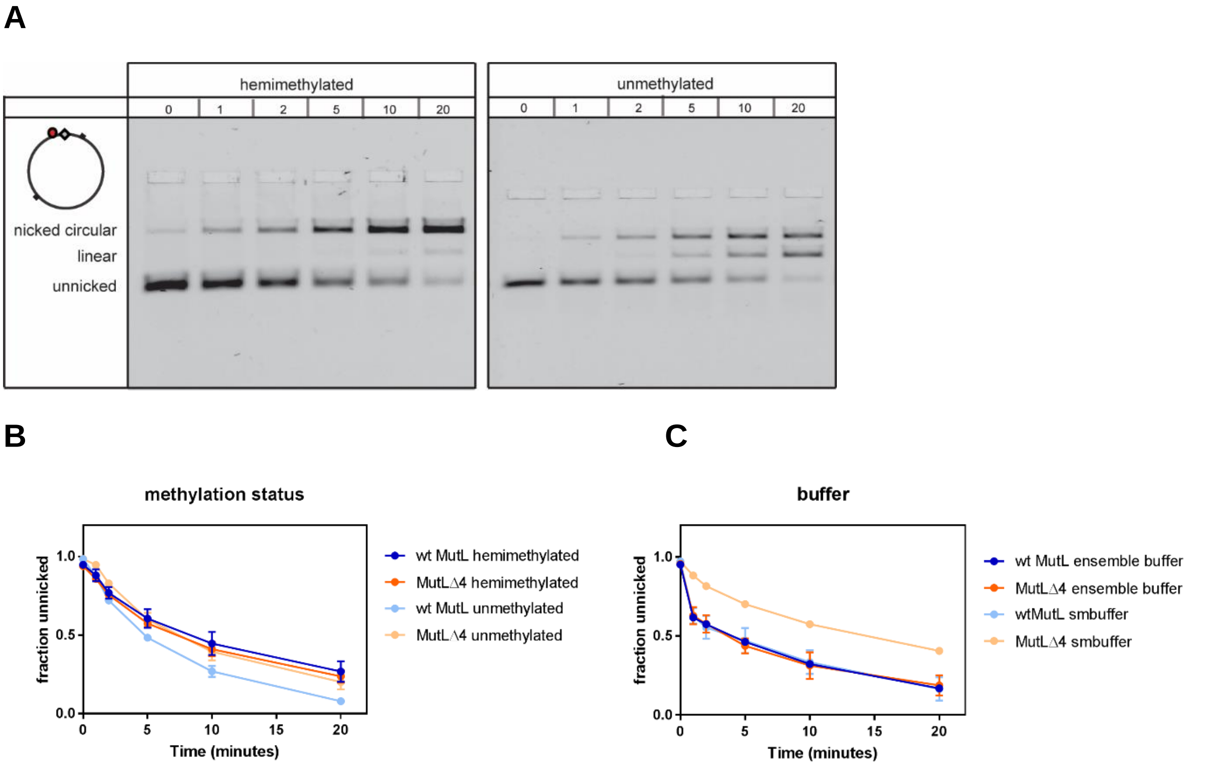

Supplemental Figure S5: Comparison of ensemble and single molecule assay conditions.

A) Similar incision rates for GATC sites on hemimethylated and unmethylated DNA. Time course of incision of 0.5 nM circular hemimethylated and unmethylated GT#2 by 2.5 nM MutS, 10 nM MutL, 10 nM MutH, 0.1 mM ATP. Reaction products were separated using native agarose gel electrophoresis in the presence of chloroquine and visualized using the Alexa^{647} fluorophore. The rate of disappearance of unnicked circular DNA is similar for hemi- and un-methylated DNA substrates. On unmethylated DNA linear product accumulates due to incision of the GATC site on both strands. B) GATC site incision is at least as efficient for wild type and MutLΔ4 on unmethylated as on hemimethylated DNA. C) **MutLΔ4 is slower in activating strand incision than wild type MutL in the buffer used for single molecule analysis.** Comparison of incision activities by wild type and Δ4 MutL in ensemble and single molecule reaction buffer (containing 0.1 mg/ml BSA, 0.005% Tween, 10% glycerol and 1 mg/ml BSA, 0.05% Tween, no glycerol, respectively). MutS and MutL were preloaded in the presence of ATP before MutH addition to mimic single molecule reaction conditions.
